# A new species of *Jesogammarus* from the Iki Island, Japan (Crustacea, Amphipoda, Anisogammaridae)

**DOI:** 10.3897/zookeys.530.6063

**Published:** 2015-10-28

**Authors:** Ko Tomikawa

**Affiliations:** 1 Department of Science Education, Graduate School of Education, Hiroshima University, Higashi-Hiroshima 739-8524, Japan

**Keywords:** *Jesogammarus*, Anisogammaridae, Amphipoda, Iki Island, Japan, new species, taxonomy

## Abstract

A new species of anisogammarid amphipod, Jesogammarus (Jesogammarus) ikiensis **sp. n.**, is described from freshwaters in the Iki Island, Nagasaki Prefecture, Japan, based on results of morphological and molecular analyses. The new species is distinguished from all members of the genus by the combination of small number of setae on dorsal margins of pleonites 1–3, short and small number of setae on posterior margins of peduncular articles of antennae, mandibular article 1 without setae, well developed posterior lobes of accessory lobes of coxal gills on gnathopod 2 and pereopods 3–5, and pectinate setae on palmar margin of female gnathopod 2. A key to all the species of *Jesogammarus* is provided.

## Introduction

The amphipod genus *Jesogammarus* Bousfield, 1979 has been recorded from fresh and brackish waters of the Japanese archipelago, the Korea peninsula, and the Chinese continent (Bousfield 1979; Morino 1984, 1985, 1986, 1993; Lee and Seo 1990, 1992; Tomikawa and Morino 2003; Tomikawa et al. 2003, Hou and Li 2004, 2005). To date, 17 species in two subgenera, *Jesogammarus* Bousfield, 1979 and *Annanogammarus* Bousfield, 1979, have been recognized.

**Figure 1.**
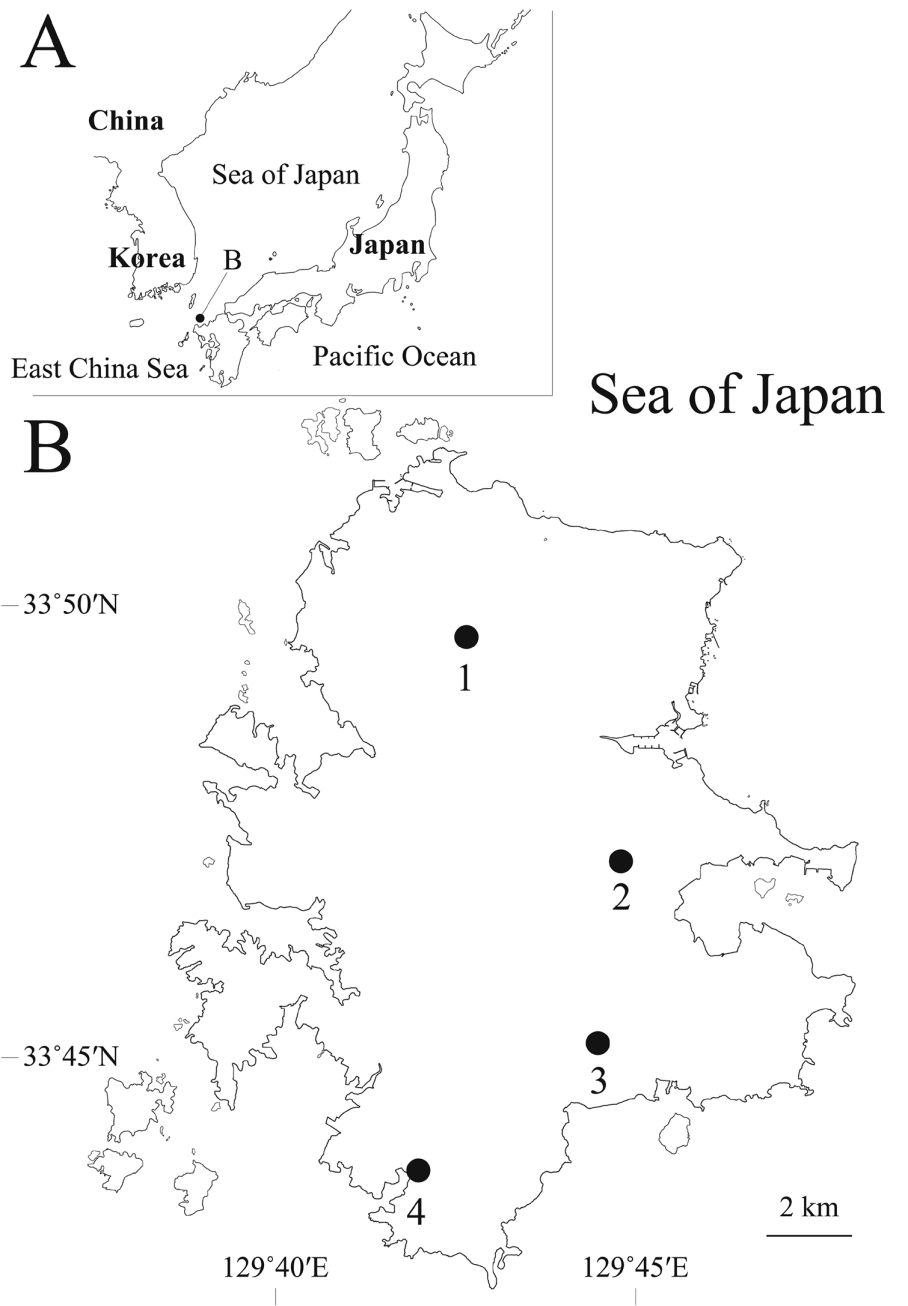
Sampling localities for Jesogammarus (Jesogammarus) ikiensis sp. n. **A** Map of Japan and adjacent area showing Iki Island, Nagasaki Prefecture, Japan **B** the collecting localities of Iki Island: 1, Katsumoto; 2, Ashibe; 3, Ishida; 4, Gonoura.

In 2010, Mr. Y. Tohyama of Hiroshima University provided a few specimens of freshwater amphipod collected from the Iki Island, Nagasaki Prefecture, Japan. They proved to belong to a previously unknown species of *Jesogammarus*. The Iki Island is located between Kyushu and the Tsushima Island, and 14 km from east to west and 17 km from north to south (Fig. 1). During field surveys of freshwater amphipods in the Iki Island, made in 2010–2015, a significant number of specimens of this species have been accumulated. Close examination of the external morphology and molecular analyses based on mitochondrial DNA sequences revealed that the Iki species is distinct from its congeners, and it is described as a new species.

## Materials and methods

### Samples

Specimens of *Jesogammarus ikiensis* sp. n. were collected from four localities in Iki Island, Nagasaki Prefecture, Japan (Fig. 1) by scooping with a fine-mesh hand-net, and preserved in 99% ethanol at the sites. For comparison, DNA sequences data were obtained for specimens of all the Japanese species of *Jesogammarus*, Jesogammarus (Annanogammarus) annandalei (Tattersall, 1922), Jesogammarus (Annanogammarus) fluvialis Morino, 1985, Jesogammarus (Jesogammarus) fujinoi Tomikawa & Morino, 2003, Jesogammarus (Jesogammarus) hinumensis Morino, 1993, Jesogammarus (Jesogammarus) hokurikuensis Morino, 1985, Jesogammarus (Jesogammarus) jesoensis (Schellenberg, 1937), Jesogammarus (Jesogammarus) mikadoi Tomikawa, Morino & Mawatari, 2003, Jesogammarus (Annanogammarus) naritai Morino, 1985, Jesogammarus (Jesogammarus) paucisetulosus Morino, 1984, Jesogammarus (Jesogammarus) shonaiensis Tomikawa & Morino, 2003, Jesogammarus (Jesogammarus) spinopalpus Morino, 1985, and Jesogammarus (Annanogammarus) suwaensis Morino, 1986. Details of these specimens are shown in Table 1. No sequence data are available for Jesogammarus (Annanogammarus) debilis Hou & Li, 2005, Jesogammarus (Jesogammarus) fontanus Hou & Li, 2004, Jesogammarus (Jesogammarus) hebeiensis Hou & Li, 2004 Jesogammarus (Jesogammarus) ilhoii Lee & Seo, 1990, or Jesogammarus (Annanogammarus) koreaensis Lee & Seo, 1992.

**Table 1.** Species, sampling localities, and numbers of specimens used for molecular phylogenetic study.

Species	Voucher	Locality	DDBJ Acc. No.		Reference
			COI	16S	
*Eogammarus kygi*	G1	Naibetsu River, Eniwa, Hokkaido, Japan	LC052229	LC052250	this study
*Eogammarus possjeticus*	G3	Akkeshi, Hokkaido, Japan	LC052230	LC052251	this study
*Jesogammarus annandalei*	G1162	Lake Biwa, Shiga Prefecture, Japan	LC052231	LC052252	this study
*Jesogammarus fluvialis*	G83	Samegai, Shiga Prefecture, Japan	LC052232	LC052253	this study
*Jesogammarus fujinoi*	G17	Gobanmiki, Yamagata, Yamagata Prefecture, Japan	LC052233	LC052254	this study
*Jesogammarus hinumensis*	G52	Lake Hinuma, Ibaraki Prefecture, Japan	LC052234	LC052255	this study
*Jesogammarus hokurikuensis*	G383	Takinami, Fukui, Fukui Prefecture, Japan	LC052235	LC052256	this study
*Jesogammarus jesoensis*	G164	Sapporo, Hokkaido, Japan	LC052236	LC052257	this study
*Jesogammarus mikadoi*	G13	Rokugo, Akita Prefecture, Japan	LC052237	LC052258	this study
*Jesogammarus naritai*	G1167	Lake Biwa, Shiga Prefecture, Japan	LC052238	LC052259	this study
*Jesogammarus paucistulosus*	G1037	Mito, Ibaraki Prefecture, Japan	LC052239	LC052260	this study
*Jesogammarus shonaiensis*	G192	Sakata, Yamagata Prefecture, Japan	LC052240	LC052261	this study
*Jesogammarus ikiensis* sp. n.	G515	Katsumoto, Iki, Nagasaki Prefecture, Japan	LC052241	LC052262	this study
*Jesogammarus ikiensis* sp. n.	G665	Ishida, Iki, Nagasaki Prefecture, Japan	LC052242	LC052263	this study
*Jesogammarus ikiensis* sp. n.	G695	Ishida, Iki, Nagasaki Prefecture, Japan	LC052243	LC052264	this study
*Jesogammarus ikiensis* sp. n.	G885	Ishida, Iki, Nagasaki Prefecture, Japan	LC052244	LC052265	this study
*Jesogammarus ikiensis* sp. n.	G886	Ishida, Iki, Nagasaki Prefecture, Japan	LC052245	LC052266	this study
*Jesogammarus spinopalpus*	G32	Onjuku, Chiba Prefecture, Japan	LC052246	LC052267	this study
*Jesogammarus suwaensis*	G88	Lake Suwa, Nagano Prefecture, Japan	LC052247	LC052268	this study
*Jesogammarus suwaensis*	G89	Lake Suwa, Nagano Prefecture, Japan	LC052248	LC052269	this study
*Spasskogammarus spasskii*	G35	Akkeshi, Hokkaido, Japan	LC052249	LC052270	this study

### Morphological observation

All appendages of the examined specimens of *Jesogammarus ikiensis* sp. n. were dissected in 99% ethanol and mounted in gum-chloral medium on glass slides under a stereomicroscope (Olympus SZX7). Specimens were examined using a light microscope (Nikon Eclipse Ni) and illustrated with the aid of a camera lucida. The body length from the tip of the rostrum to the base of the telson was measured along the dorsal curvature to the nearest 0.1 mm. The nomenclature of the setal patterns on the mandibular palp follows Stock (1974). The specimens are deposited in the Tsukuba Collection Center of the National Museum of Nature and Science, Tokyo (NSMT).

### DNA extraction, PCR amplification, and DNA sequencing

Total genomic DNA was extracted from pereopod musculature of each sequenced amphipod (Table 1), by means of the DNeasy blood and tissue kit (Qiagen, Hilden, Germany); the final volume of the DNA solution following extraction was 200 µl. Part of the mitochondrial cytochrome *c* oxidase subunit I (COI) and 16S ribosomal RNA (rRNA) genes were amplified by polymerase chain reaction (PCR) using the following primer pair: Am-COI-H [CG(AG)GC(CGT)TA(CT)TT(CT)AC(CT)TC(ATC)GC(AC)ACTAT] and Am-COI-T [CGTCG(AGT)GG(CT)AT(ACG)CC(ACGT)CT(AGT)A(AG)(ATC)CCTA] (Tomikawa et al. 2007); 16STf [GGTAA(T)A(CT)C(T)TA(G)ACC(T)GTGCTAAG] (Macdonald et al. 2005) and 16Sbr [CCGGTTTGAACTCAGATCATGT] (Palumbi et al. 1991). PCR reactions containing 0.5 µl template solution, 2 mM MgCl_2_, 2.5 mM dNTP, 10 pmol of each primer, and 5U/µl Taq polymerase (TaKaRa Ex Taq®) in 1X buffer provided by the manufacturer were performed in 10-µl volumes in an PC-320 thermal cycler (ASTEC). Amplification conditions were as follows: an initial denaturation for 7 min at 94 °C; 35 cycles of denaturation for 45 s at 94 °C, annealing for 1 min at 42–50 °C depending on samples, and extension for 1 min at 72 °C; and final extension for 7 min at 72 °C. Amplification products were purified by the silica method (Boom et al. 1990). All sequencing reactions were performed according to the manufacturer’s instructions using the BigDye Terminater v3.1 Cycle Sequencing Reaction Kit (Applied Biosystems, Foster City, CA). Cycle sequencing conditions were 25 cycles of 10 s at 96 °C, 5 s at 50 °C, and 4 min at 60 °C. Sequencing reaction products were purified by ethanol precipitation. Labeled fragments were analyzed using an ABI 3130x Genetic Analyzer (Applied Biosystem). Sequences were obtained from both strands of the gene segments for verification using the same primers. The nucleotide sequences have been submitted to the DNA Databank of Japan (DDBJ) nucleotide-sequence database (linked to the EMBL and GenBank databases) (Table 1).

### Molecular phylogenetic analyses

The nucleotide sequences were aligned using the multiple alignment algorithm in Clustal W (Thompson et al. 1994) with default setting (i.e., gap opening penalty = 15, gap extension penalty = 6.66, transition weight = 0.5). Phylogenetic relationships were reconstructed by the Neighbor-Joining method (NJ; Saitou and Nei 1987), the equally weighted maximum parsimony method (MP), and the maximum likelihood method (ML) with MEGA6 software (Tamura et al. 2013). There was no indel in COI sequences of the ingroup taxa. On the other hand, eight indels were found in 16S sequences of the ingroup taxa, which were treated as missing data in all analyses. In the NJ analysis, the Kimura 2-parameter (K2P) model (Kimura 1980) of nucleotide substitution was used to estimate genetic distances. In the MP analysis, a tree was obtained using the Close-Neighbor-Interchange algorithm, in which the initial trees were obtained with the random addition of sequences (10 replicates). The ML analysis used the T92 + G + I model for COI and HKY + G for 16S and COI + 16S; this was selected as the best-fit model using the Bayesian information criterion (BIC) in MEGA6. To estimate statistical support for branching patterns, 1,000 bootstrap replications each (Felsenstein 1985) were performed for the NJ, MP, and ML analyses. As outgroup taxa, three anisogammarid species, *Eogammarus kygi* (Derzhavin, 1923), *Eogammarus possjeticus* (Tzvetkova, 1967), and *Spasskogammarus spasskii* (Bulycheva, 1952), were used (Table 1).

### Remarks

**Figure 2.**
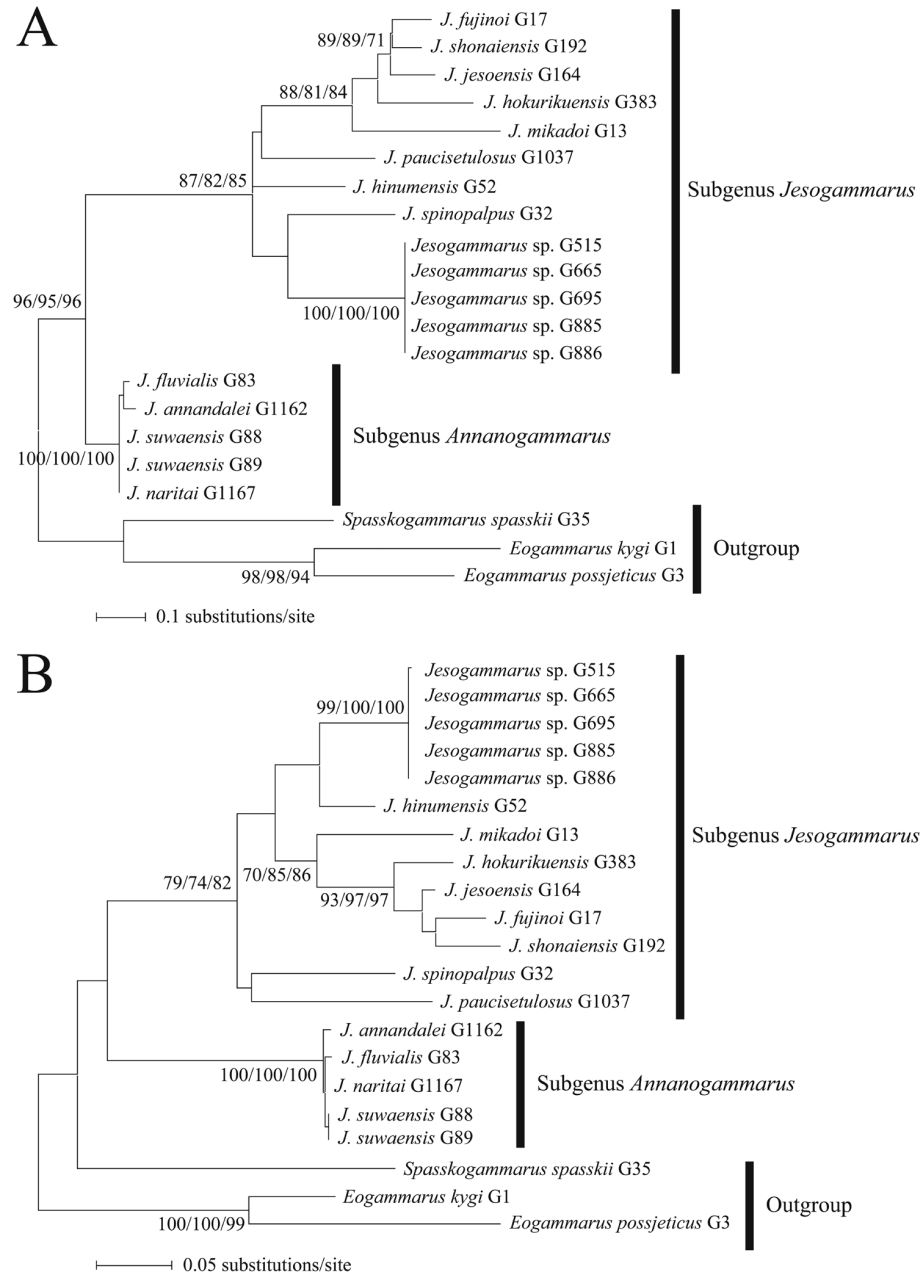
**A** maximum-likelihood tree from a 333 bp sequence of COI gene **B** maximum-likelihood tree from a 416 bp sequence of 16S rRNA gene. Numbers near the branches are ML/NJ/MP bootstrap values. Bootstraps are shown when ≥ 70%. Vouchers are shown after species names as in Table 1.

**Figure 3.**
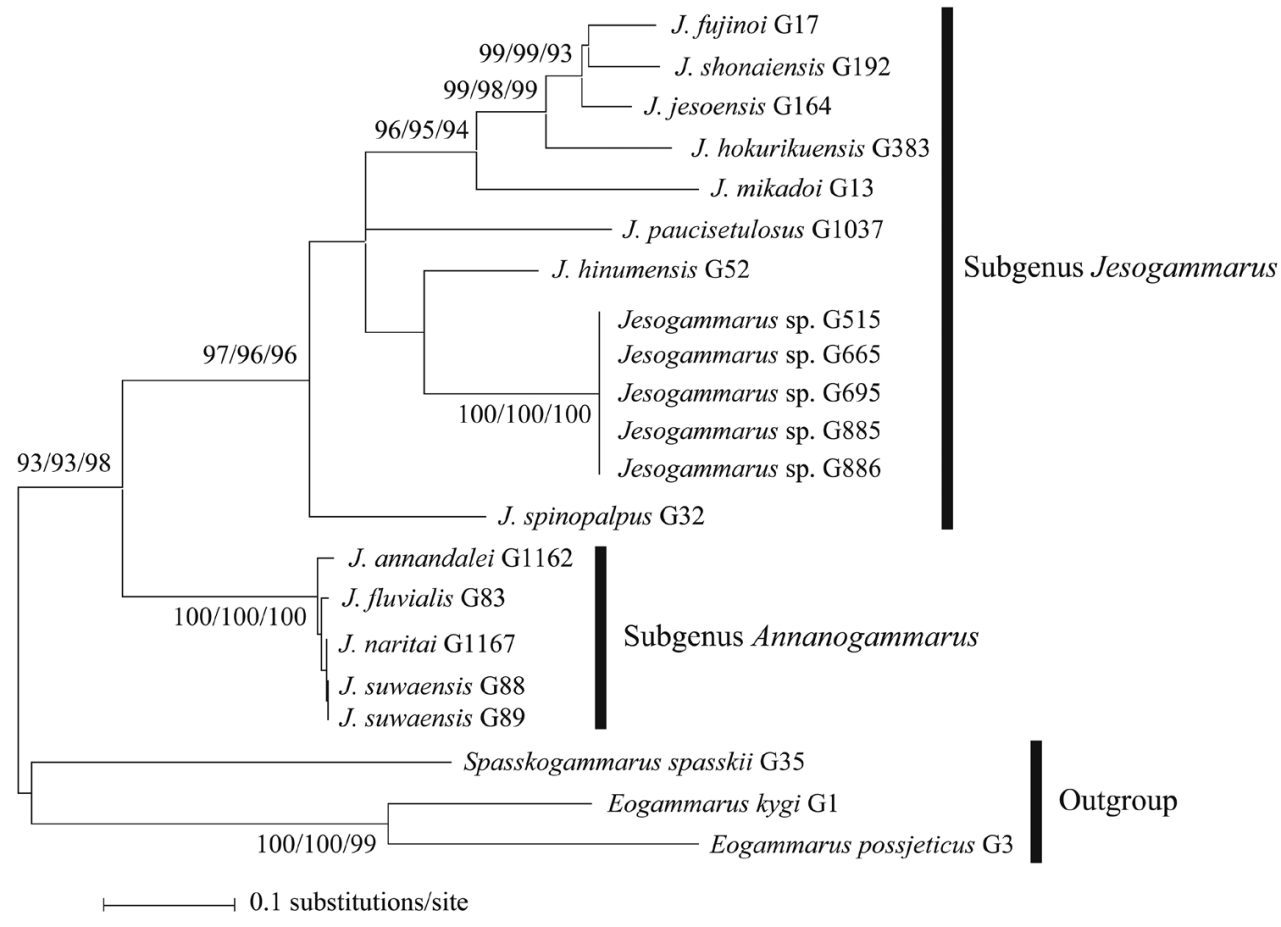
Maximum-likelihood tree from a 749 bp sequence of COI + 16S rRNA genes. Numbers near the branches are ML/NJ/MP bootstrap values. Bootstraps are shown when ≥ 70%. Vouchers are shown after species names as in Table 1.

Monophyly of the subgenera *Jesogammarus* and *Annanogammarus* were supported in COI, 16S, and COI + 16S trees (Figs 2, 3). *Jesogammarus ikiensis* sp. n. from Iki Island was included in the clade of the subgenus *Jesogammarus*. However, phylogenetic position of *Jesogammarus ikiensis* was not clearly resolved in the phylogenetic trees based on the COI and 16S rRNA genes due to low bootstrap values. *Jesogammarus ikiensis* differs from the all Japanese congeners by large genetic distances (18.6–25.8% for COI and 12.7–18.7% for 16S) (Table 2), which were larger than intraspecific distances among many species of *Jesogammarus*. In addition, *Jesogammarus ikiensis* was morphologically distinguished from its congeners. Thus, it can be concluded that *Jesogammarus ikiensis* from Iki Island as a distinct new species and is described below.

**Table 2.** Uncorrected pairwise differences (%: p-distance) of partial COI (upper right) and 16S rRNA (lower left) gene sequences between species.

	1	2	3	4	5	6	7	8	9	10	11	12	13	14	15	16
1: *Eogammarus kygi* (N = 1)		19.5	24.3	23.7	30.6	27.3	27.0	27.9	30.0	24.3	28.5	29.1	**29.4**	27.6	24.3	25.2
2: *Eogammarus possjeticus* (N = 1)	16.3		24.9	24.0	27.0	27.0	26.4	25.5	27.6	23.7	28.5	27.6	**28.5**	28.2	23.4	24.3
3: *Jesogammarus annandalei* (N = 1)	20.8	22.3		3.0	24.0	20.7	23.1	22.5	25.8	3.3	20.7	24.3	**21.6**	20.7	3.0–3.3	22.5
4: *Jesogammarus fluvialis* (N = 1)	20.4	22.0	2.0		24.0	19.5	22.5	21.9	25.2	2.1	20.7	22.8	**21.6**	20.4	1.8–2.1	22.8
5: *Jesogammarus fujinoi* (N = 1)	25.1	24.0	20.6	20.7		19.8	15.3	10.5	18.9	22.5	20.4	9.3	**24.6**	21.3	22.8–23.1	27.9
6: *Jesogammarus hinumensis* (N = 1)	22.3	23.9	18.0	17.6	15.1		20.7	17.7	21.9	19.5	18.6	18.6	**19.5**	18.6	19.2–19.5	25.8
7: *Jesogammarus hokurikuensis* (N = 1)	24.3	24.3	20.3	20.2	11.2	15.6		17.1	21.3	22.5	19.5	12.6	**23.7**	19.8	22.2–22.5	26.1
8: *Jesogammarus jesoensis* (N = 1)	24.2	23.8	20.2	20.0	6.9	13.6	10.8		18.6	21.6	20.1	10.5	**25.8**	21.0	21.0–21.3	28.2
9: *Jesogammarus mikadoi* (N = 1)	25.1	24.7	21.0	20.7	15.4	16.4	15.6	14.3		24.0	20.4	18.9	**25.8**	21.6	23.7–24.0	28.5
10: *Jesogammarus naritai* (N = 1)	20.8	21.6	1.9	1.2	19.8	17.5	19.9	19.6	20.0		20.1	22.2	**20.7**	20.1	0.3–0.6	23.4
11: *Jesogammarus paucisetulosus* (N = 1)	24.2	25.2	18.7	18.8	16.3	15.6	16.3	16.4	18.7	18.3		19.8	**21.0**	21.0	20.1–20.7	25.2
12: *Jesogammarus shonaiensis* (N = 1)	25.5	25.1	21.9	21.4	7.3	15.5	10.3	7.3	15.1	20.8	17.4		**22.8**	20.4	21.9–22.2	27.9
13: ***Jesogammarus ikiensis* sp. n.** (N = 5)	**23.4–23.5**	**24.0–24.2**	**18.2–18.3**	**18.3–18.4**	**18.0–18.2**	**12.7–12.8**	**17.8–17.9**	**18.2–18.3**	**18.6–18.7**	**17.6–17.8**	**16.8–17.0**	**17.4**		**18.6**	**20.4–20.7**	**26.1**
14: *Jesogammarus spinopalpus* (N = 1)	23.0	24.6	17.0	17.0	17.1	15.9	16.6	16.7	16.8	16.6	16.4	17.1	**14.8–15.0**		19.5–19.8	24.6
15: *Jesogammarus suwaensis* (N = 2)	20.8–21.0	21.6–21.8	1.9–2.0	1.2–1.3	20.0–20.2	17.5–17.6	20.0–20.2	19.5–19.6	20.0–20.2	0.3–0.4	18.3–18.4	20.8–21.0	**17.6–17.9**	16.4–16.6		22.8–23.1
16: *Spasskogammarus spasskii* (N = 1)	22.2	22.8	20.6	20.8	22.7	22.0	22.2	22.3	23.0	20.8	21.1	22.4	**21.5–21.6**	21.0	20.6–20.7	

## Systematics

### Jesogammarus (Jesogammarus) ikiensis sp. n.

Taxon classificationAnimaliaAmphipodaAnisogammaridae

http://zoobank.org/75FDE441-CD57-41C4-B154-1E3D29ECAC3E

New Japanese name: Iki-yokoebi

**Figure 4.**
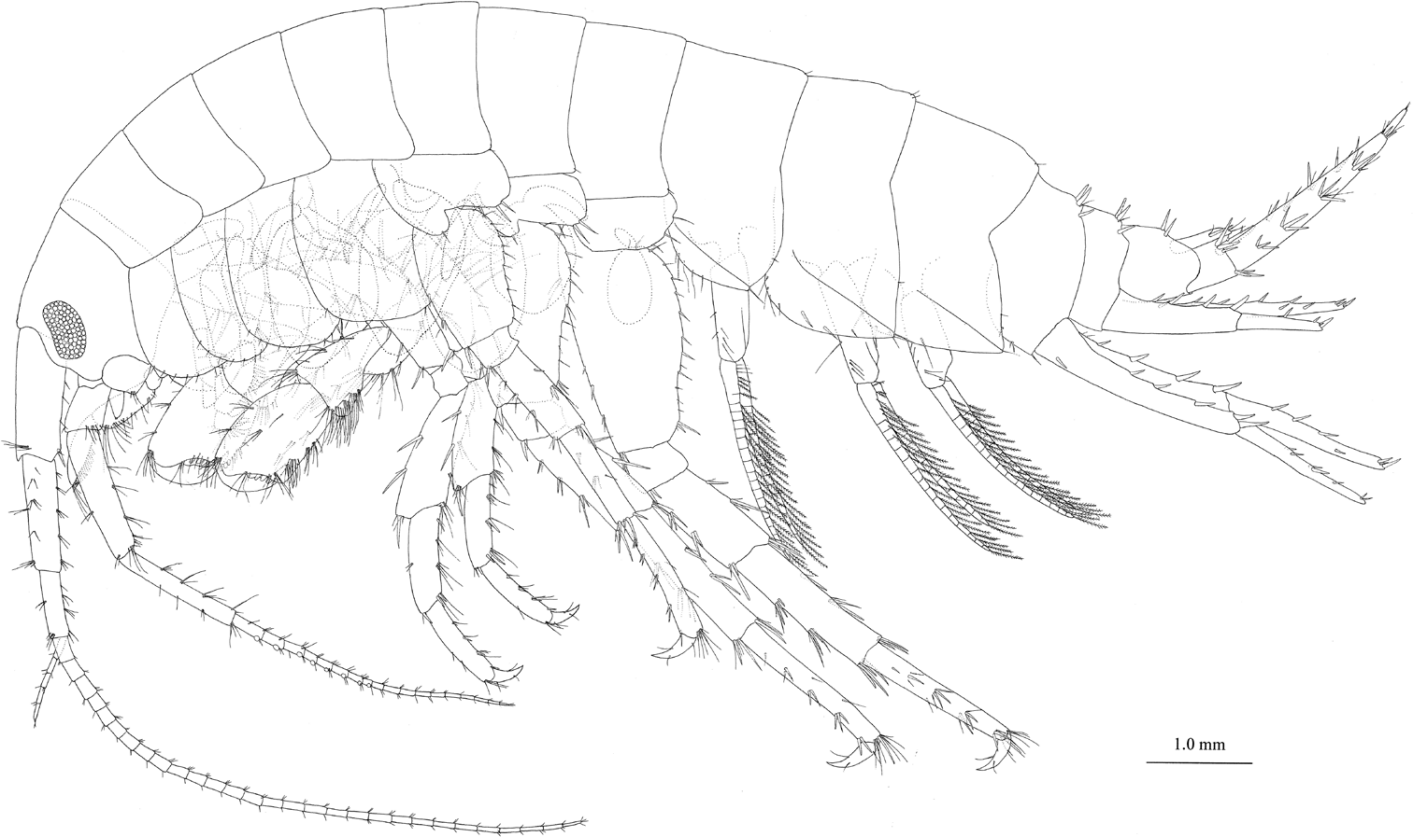
Jesogammarus (Jesogammarus) ikiensis sp. n., holotype, male, 13.1 mm, NSMT-Cr 24107, Ishida, Iki, Nagasaki Prefecture, Japan. *Habitus*, lateral view.

**Figure 5.**
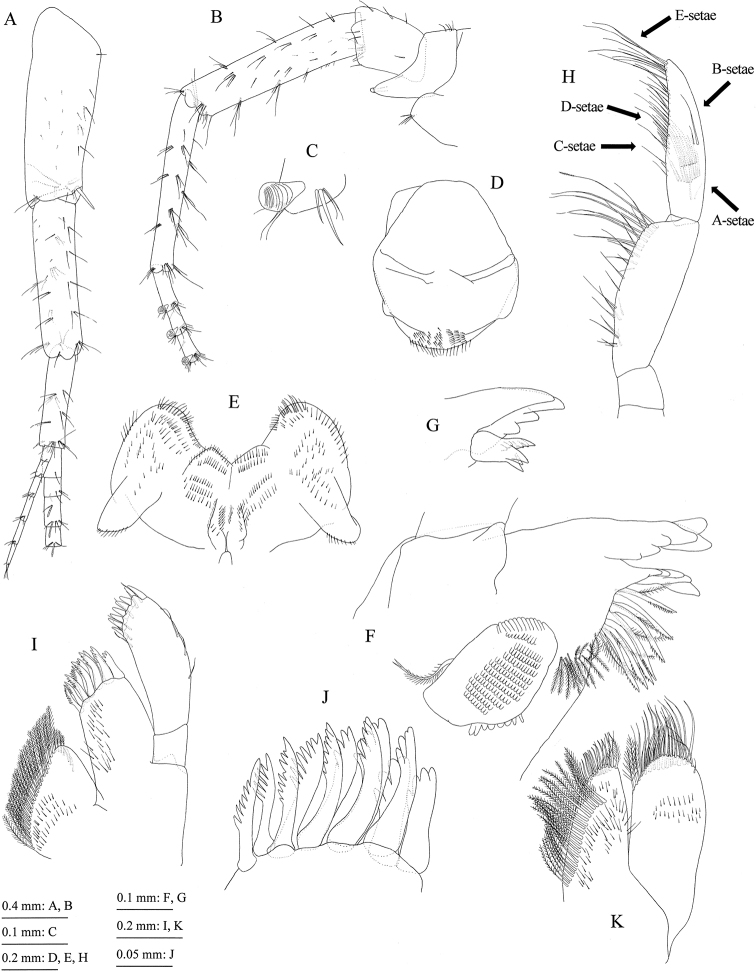
Jesogammarus (Jesogammarus) ikiensis sp. n., holotype, male, 13.1 mm, NSMT-Cr 24107, Ishida, Iki, Nagasaki Prefecture, Japan. **A** peduncular articles 1–3, accessory flagellum, and flagellar articles 1–4 of antenna 1, medial view (posterio-marginal setae on peduncular articles 2 and 3 indicated by arrowheads) **B** peduncular articles 1–5 and flagellar articles 1–3 of antenna 2, medial view (posterio-marginal setae on peduncular articles 4 and 5 indicated by arrowheads) **C** calceolus of antenna 2, medial view **D** upper lip, anterior view **E** lower lip, ventral view **F** left mandible except palp, medial view **G** incisor and lacinia mobilis of right mandible, lateral view **H** palp of right mandible, medial view **I** maxilla 1, dorsal view **J** outer plate of maxilla 1, dorsal view **K** Maxilla 2, dorsal view.

**Figure 6.**
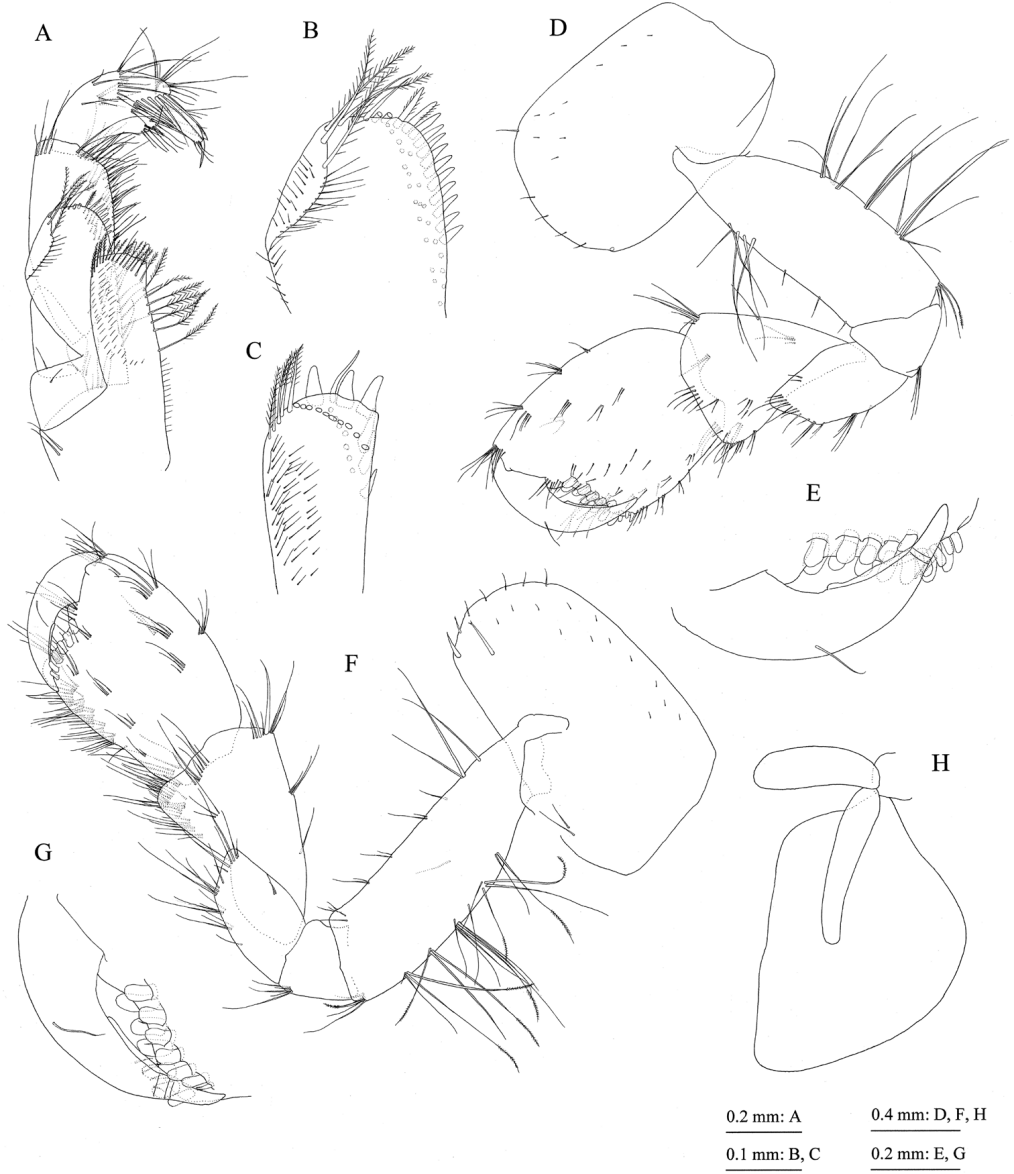
Jesogammarus (Jesogammarus) ikiensis sp. n., holotype, male, 13.1 mm, NSMT-Cr 24107, Ishida, Iki, Nagasaki Prefecture, Japan. **A** maxilliped, dorsal view **B** outer plate of maxilliped, dorsal view **C** inner plate of maxilliped, dorsal view **D** gnathopod 1, medial view **E** palmar margin of propodus and dactylus of gnathopod 1, medial view **F** gnathopod 2, medial view **G** palmar margin of propodus and dactylus of gnathopod 2, medial view **H** coxal gill of gnathopod 2, medial view.

**Figure 7.**
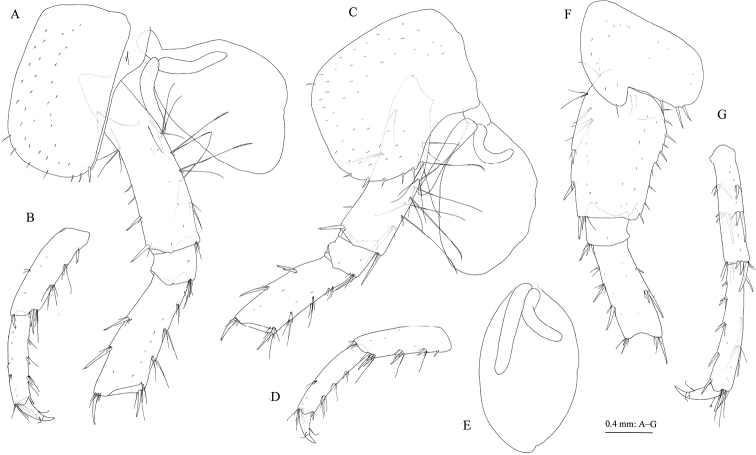
Jesogammarus (Jesogammarus) ikiensis sp. n., holotype, male, 13.1 mm, NSMT-Cr 24107, Ishida, Iki, Nagasaki Prefecture, Japan. **A** coxa–merus and coxal gill of pereopod 3, lateral view **B** carpus–dactylus of pereopod 3, lateral view **C** coxa–merus and coxal gill of pereopod 4, lateral view **D** carpus–dactylus of pereopod 4, lateral view **E** coxal gill of pereopod 5, lateral view **F** coxa–merus of pereopod 5, lateral view **G** carpus–dactylus of pereopod 5, lateral view.

**Figure 8.**
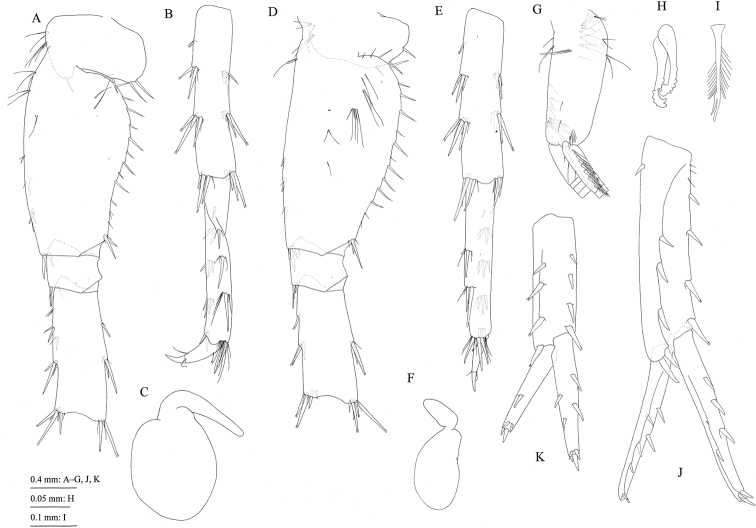
Jesogammarus (Jesogammarus) ikiensis sp. n., holotype, male, 13.1 mm, NSMT-Cr 24107, Ishida, Iki, Nagasaki Prefecture, Japan. **A** coxa–merus of pereopod 6, lateral view **B** carpus–dactylus of pereopod 6, lateral view **C** coxal gill of pereopod 6, lateral view **D** coxa–merus of pereopod 6, lateral view **E** carpus–dactylus of pereopod 6, lateral view **F** coxal gill of pereopod 7, lateral view **G** pleopod 1, medial view, distal parts of rami omitted **H** retinacula on peduncle of pleopod 1, medial view **I** bifid plumose seta (clothes-pin seta) on inner basal margin of inner ramus of pleopod 1, medial view **J** uropod 1, dorsal view **K** uropod 2, dorsal view.

**Figure 9.**
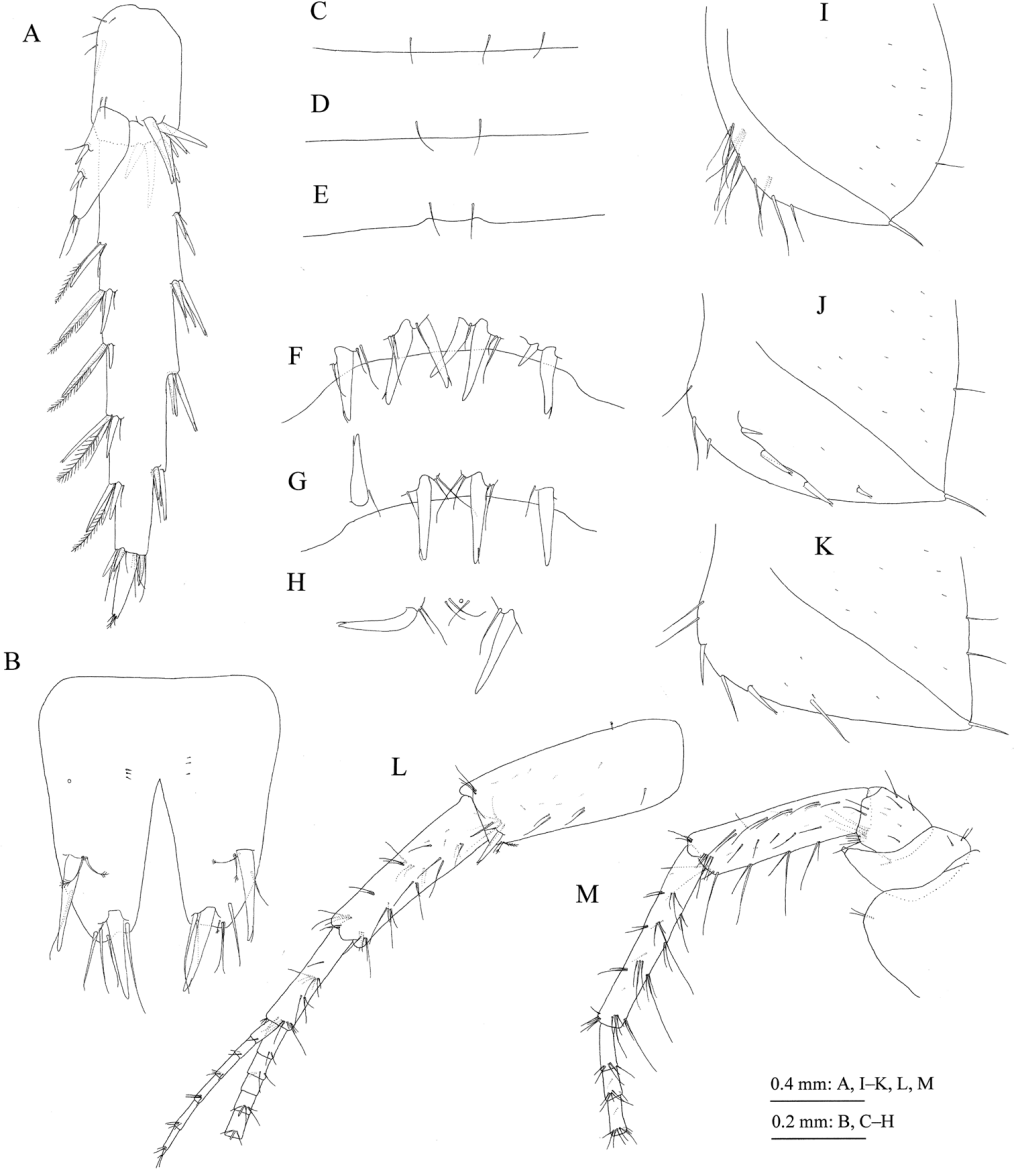
Jesogammarus (Jesogammarus) ikiensis sp. n., Ishida, Iki, Nagasaki Prefecture, Japan. Holotype, male, 13.1 mm, NSMT-Cr 24107 (A–K) and paratype, female, 10.4 mm, NSMT-Cr 24108 (L and M). **A** uropod 3, dorsal view **B** telson, dorsal view **C–E** pleonites 1–3, respectively, dorsal views **F–H** urosomites 1–3, respectively, dorsal views **I–K** epimeral plates 1–3, respectively, lateral views **L** peduncular articles 1–3, accessory flagellum, and flagellar articles 1–5 of antenna 1, medial view **M** peduncular articles 1–5 and flagellar articles 1–3 of antenna 2, medial view.

Figures 3 , 4 , 5 , 6 , 7 , 8 , 9

#### Material examined.

Holotype: NSMT-Cr 24107, male (13.1 mm, 8 slides), river at Ishida (33°45'1.7"N, 129°44'33.7"E), Iki, Nagasaki Prefecture, Japan, collected by K. Tomikawa and S. Tashiro on 9 March 2012. Paratypes: NSMT-Cr 24108, ovigerous female (10.4 mm, 6 slides), NSMT-Cr 24109, 1 male and 1 ovigerous female in ethanol vial, data same as for holotype; NSMT-Cr 24110, 2 males and 2 ovigerous females in ethanol vial, river at Ishida (33°45'1.7"N, 129°44'33.7"E), Iki, Nagasaki Prefecture, Japan, collected by K. Tomikawa and S. Tashiro on 2 April 2015; NSMT-Cr 24111, male (12.0 mm, 6 slides), NSMT-Cr 24112, ovigerous female (9.4 mm, 5 slides), river at Katsumoto (33°49'30.1"N, 129°42'51.5"E), Iki, Nagasaki Prefecture, Japan, collected by K. Tomikawa and S. Tashiro on 8 March 2012; NSMT-Cr 24113, male (11.9 mm, 5 slides), NSMT-Cr 24114, ovigerous female (10.0 mm, 5 slides), river at Ashibe (33°47'3.1"N, 129°45'3.8"E), Iki, Nagasaki Prefecture, Japan, collected by K. Tomikawa and S. Tashiro on 9 March 2012; NSMT-Cr 24115, male (9.2 mm, 5 slides), NSMT-Cr 24116, female with offsprings (7.4 mm, 5 slides), irrigation ditch at Gonoura (33°43'26"N, 129°41'52"E), Iki, Nagasaki Prefecture, Japan, collected by K. Tomikawa and S. Tashiro on 9 March 2012.

#### Description of male

**(holotype, NSMT-Cr** 24107). Head (Fig. 4) with short rostrum; ventral margin of lateral cephalic lobe weakly concave; antennal sinus rounded; eyes reniform, major axis 0.4 × height of head. Dorsal surfaces of pereonites smooth (Fig. 4). Dorsal margins of pleonites 1–3 (Fig. 9C–E) with three, two, and two setae, respectively. Posterior margin of epimeral plate 1 rounded with seta, anteroventral corner with many setae (Fig. 9I); posterior margin of plate 2 with one seta, posteroventral corner quadrate, anteroventral corner with three setae, ventral submargin with four robust setae (Fig. 9J); posterior margin of plate 3 with two setae, posteroventral corner quadrate, anteroventral to ventral margin with six setae (Fig. 9K). Urosomites 1–3 (Fig. 9F–H) with seven, four, and two robust setae associated with slender setae.

*Antenna 1* (Fig. 5A): length 0.7 × body length; peduncular articles 1–3 in length ratio of 1.0 : 0.9 : 0.5; posterodistal corner of peduncular article 1 with one robust seta, posterior margin of peduncular article 2 with one cluster and three pairs of setae, posterior margin of peduncular article 3 with one cluster and one pair of setae; accessory flagellum seven-articulate; primary flagellum 29-articulate, each article with one aesthetasc.

*Antenna 2* (Fig. 5B): length 0.7 × antenna 1; posterior margin of peduncular article 4 with three clusters of setae, posterior margin of peduncular article 5 with three clusters of setae and one single seta; flagellum 18-articulate, calceoli present (Fig. 5C).

*Mouthparts*. Upper lip (= labrum) (Fig. 5D) with rounded distal margin, bearing fine setae. Lower lip (= labium) (Fig. 5E) with broad outer lobes, inner lobes indistinct. Mandibles (Fig. 5F–H) with left and right incisors six- and four-dentate, respectively, left lacinia mobilis five-dentate, right one bifid, bearing many teeth; molar process triturative, with plumose seta; accessory setal rows of left and right mandibles each with seven blade-like setae; left palp three-articulate with length ratio of 1.0 : 3.8 : 3.8, palp article 1 bare, article 2 with 28 setae, article 3 with two clusters and one pair of A-setae, one pair of B-setae, and many C-, D-, and E-setae, article 3 of right palp with three clusters of A-seta and one B-seta. Maxilla 1 (Fig. 5I) with inner and outer plates and palp; medial margin and apical submargin of inner plate with 31 plumose setae; outer plate subrectangular, with 11 serrate teeth apically (Fig. 5J); right palp two-articulate, much longer than outer plate, article 1 lacking marginal setae, article 2 with seven robust and six slender setae on its apical margin, outer margin with three setae, left palp lacking setae on outer margin of article 2. Maxilla 2 (Fig. 5K) with oblique inner row of 23 plumose setae on inner plate; outer plate slightly longer than inner plate. Maxilliped (Fig. 6A) with inner and outer plates and palp; inner plate (Fig. 6C) with six robust setae along apical and inner margins; outer plate (Fig. 6B) with plumose setae on apical margin and robust setae on inner margin; palp four-articulate, article 2 with inner marginal and submarginal rows of setae, article 3 with facial setae, article 4 slightly curved inward, with slender nail.

*Gnathopod 1* (= pereopod 1) (Fig. 6D): coxa (= article 1) with six setae on ventral margin; anterior and posterior margins of basis (= article 2) with long setae; carpus (= article 5) length 1.4 × width, anterior margin with seta; propodus (= article 6) length 1.2 × length of carpus and 1.3 ×width of propodus, anterior margin with one pair and two clusters of setae, palmar margin (Fig. 6E) oblique, weakly convex, with 16 peg-spines (= robust setae); dactylus (= article 7) (Fig. 6E) as long as palmar margin, with posterior accessory blade longer than nail, blade basally elevated.

*Gnathopod 2* (= pereopod 2) (Fig. 6F): coxa with seven marginal and one submarginal setae on ventral part, posteroproximal part with two setae; anterior and posterior margins of basis with long setae; carpus length 1.7 × width, anterior margin with cluster of setae and single seta; propodus almost as long as carpus and 1.5 × width of propodus, anterior margin with two clusters of setae, palmar margin (Fig. 6G) oblique, weakly convex, with 12 peg-spines (= robust setae) and one serrate seta; dactylus (Fig. 6G) as long as palmar margin, with posterior accessory blade longer than nail.

*Pereopod 3* (Fig. 7A, B): coxa with seven marginal setae on ventral part, posterio-proximal part with two setae; anterior and posterior margins of basis with long setae, anterio-distal corner of basis with robust seta.

*Pereopod 4* (Fig. 7C, D): coxa expanded with posterior concavity, bearing one seta on anterodistal corner and five setae on posterodistal margin; anterior and posterior margins of basis with long setae, anterodistal corner with robust seta.

*Pereopod 5* (Fig. 7F, G): coxa bilobed, anterior lobe with apical seta, ventral margin of posterior lobe with three setae; posterior margin of basis weakly expanded, with ten setae; anterior and posterior margins of merus to propodus with robust and slender setae.

*Pereopod 6* (Fig. 8A, B): coxa bilobed, anterior lobe with apical seta and anterio-proximal setae, ventral margin of posterior lobe with three setae; posterior margin of basis weakly expanded, with 18 setae; anterior and posterior margins of merus to propodus with robust and slender setae.

*Pereopod 7* (Fig. 7D, E): ventral margin of coxa weakly concave, bearing slender setae on anterior part and three setae on posteroventral part; posterior margin of basis weakly expanded, with 20 setae; anterior and posterior margins of merus to propodus with robust and slender setae.

*Coxal gills* on gnathopod 2 and pereopods 3–5 (Figs 6H, 7A, C, E) with two accessory lobes, gills on pereopods 6 and 7 (Fig. 8C, F) each with one accessory lobe.

*Pleopods 1–3* (Fig. 8G) each with paired retinacula (Fig. 8H) on inner margin of peduncle, and bifid plumose setae (= clothes-pin setae) (Fig. 8I) on inner basal margin of inner ramus.

*Uropods*. Uropod 1 (Fig. 8J): peduncle with robust seta on basofacial part, inner and outer margins each with three robust setae, inner proximal part with three short setae; inner ramus length 0.8 × peduncle, inner margin with three robust setae and outer margin with robust seta and minute seta; outer ramus length 0.9 × inner ramus, inner and outer margins each with two and three robust setae. Uropod 2 (Fig. 8K): peduncle with three robust setae on inner and outer margins, respectively; inner ramus length 0.9 × peduncle, its inner and outer margins with two robust setae, respectively; outer ramus length 0.8 × inner ramus, its outer margin with robust seta. Uropod 3 (Fig. 9A): peduncle length 0.3 × outer ramus; inner ramus length 0.25 × outer ramus (both proximal and terminal articles), with two robust setae on inner margin; outer ramus two-articulate, inner margin of proximal article with five plumose setae, and several robust setae and simple setae, outer margin with robust setae and simple setae, terminal article length 0.2 × proximal article, with short setae apically.

*Telson* (Fig. 9B) length 1.1 × width, cleft for 59% of length in V-shape; each lobe with one lateral and one apical robust seta.

#### Description of ovigerous female

**(paratype, NSMT-Cr** 24108). Antenna 1 (Fig. 9L): length 0.7 × body length; peduncular articles 1–3 in length ratio of 1.0 : 0.8 : 0.5; accessory flagellum seven-articulate; primary flagellum 36-articulate.

*Antenna 2* (Fig. 9M): length 0.5 × antenna 1; flagellum 12-articulate, calceoli absent.

**Figure 10.**
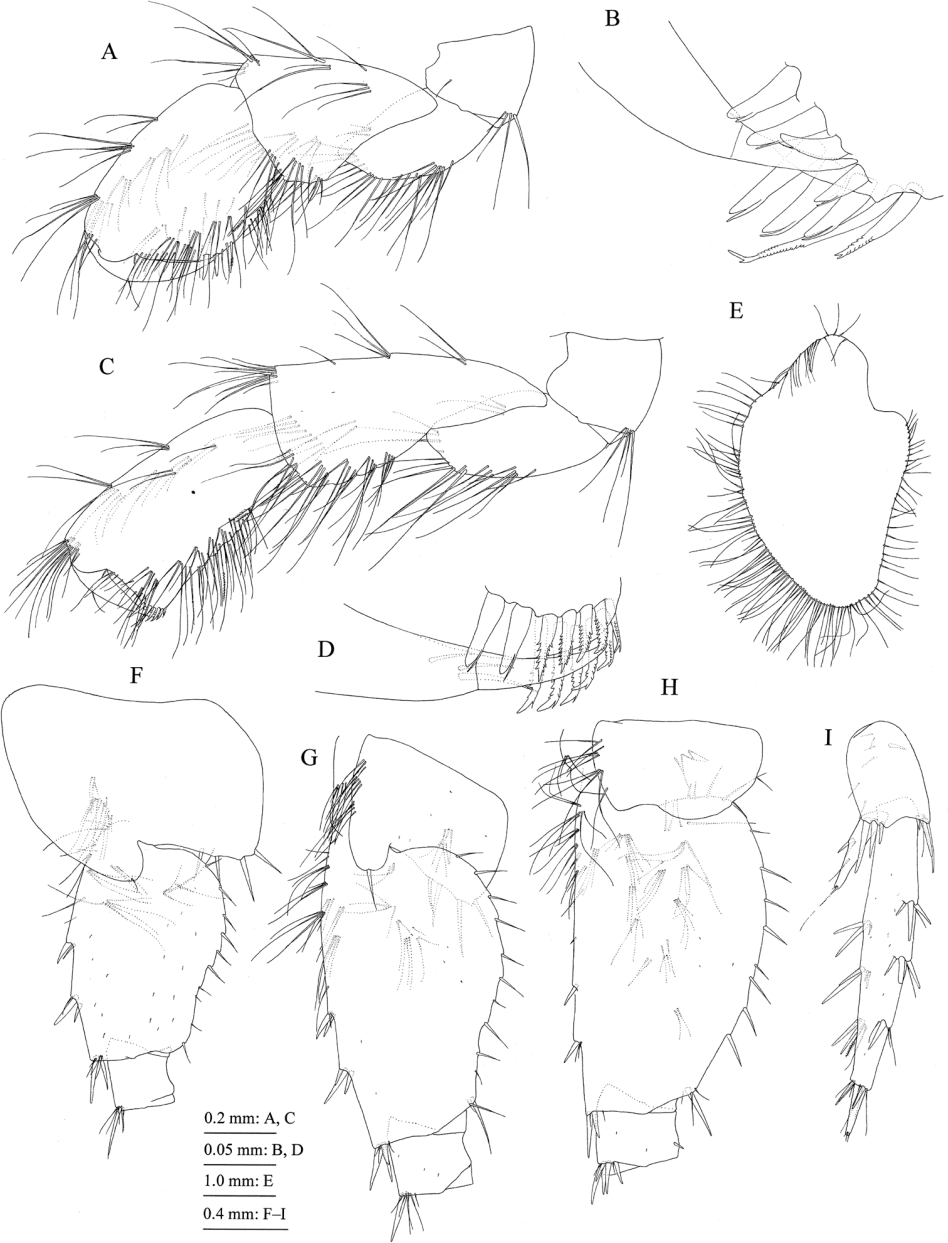
Jesogammarus (Jesogammarus) ikiensis sp. n., paratype, female, 10.4 mm, NSMT-Cr 24108, Ishida, Iki, Nagasaki Prefecture, Japan. **A** ischium–dactylus of gnathopod 1, lateral view **B** posterodistal part of palmar margin of propodus and part of dactylus of gnathopod 1, medial view **C** ischium–dactylus of gnathopod 2, lateral view **D** posterodistal part of palmar margin of propodus and part of dactylus of gnathopod 2, medial view **E** brood plate of gnathopod 2, lateral view **F** coxa–ischium of pereopod 5, lateral view **G** coxa–ischium of pereopod 6, lateral view **H** coxa–ischium of pereopod 7, lateral view **I** uropod 3, ventral view.

*Gnathopod 1* (Fig. 10A): carpus length 1.7 × width, with cluster of setae and single seta on anterior margin; propodus almost as long as carpus and 1.5 × width of propodus, bearing two clusters and one pair of setae on anterior margin; palmar margin (Fig. 10B) with seven robust setae and two pectinate setae.

**Figure 11.**
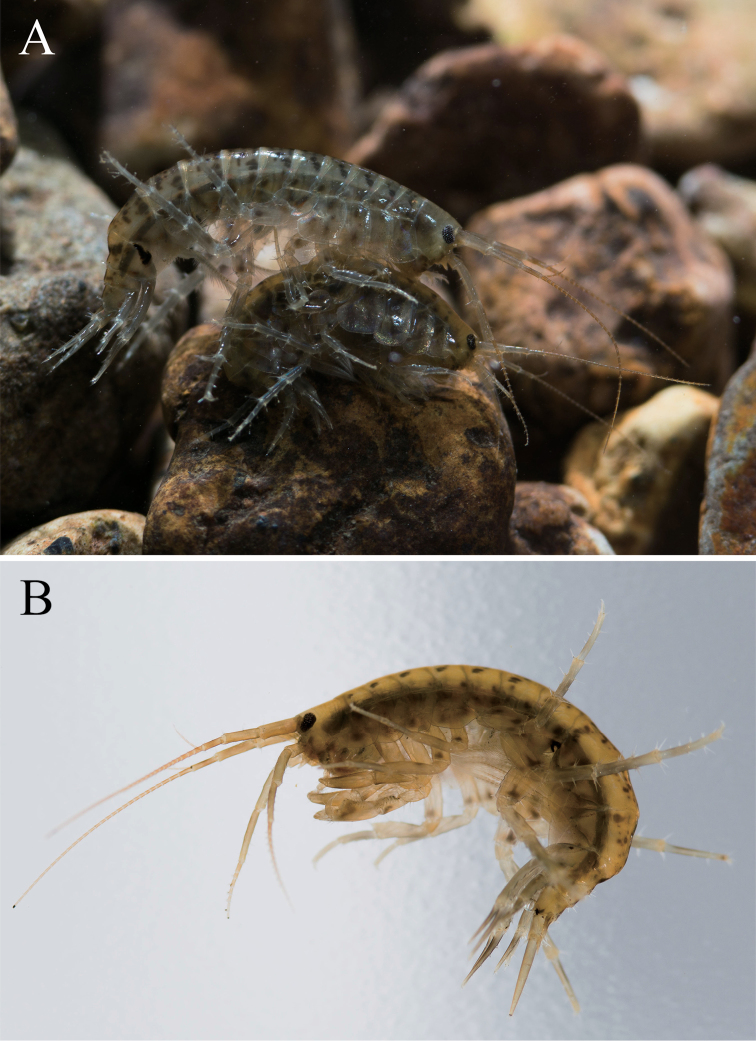
Jesogammarus (Jesogammarus) ikiensis sp. n., not preserved. **A** precopula pair (male: upper, female: lower) **B** male, approx. 13 mm. Photographed by Ryu Uchiyama.

*Gnathopod 2* (Fig. 10C): carpus length 2.2 × width, with one cluster, one pair, and one single seta on anterior margin; propodus length 0.9 and 2.0 × carpus and width of propodus, respectively, bearing one cluster and one pair of setae on anterior margin; palmar margin (Fig. 10D) with two robust and 10 pectinate setae.

Posterior margin of bases of pereopods 5–7 more expanded than in male (Fig. 10F–H).

*Brood plates* (= oostegites) (Fig. 10E): broad, with numerous marginal setae.

*Uropod 3* (Fig. 10I): peduncle length 0.3 × outer ramus; inner ramus length 0.3 × outer ramus (both proximal and terminal articles), with robust seta on inner margin; inner margin of proximal article of outer ramus with plumose seta, terminal article length 0.2 × proximal article.

Egg number: 175.

#### Variations.

The number of setae and/or setal bundles on posterior margin of peduncular articles of antennae is variable: antenna 1, two or three on article 1, three or four on article 2, one or two on article 3; antenna 2, two to four on article 4, three to five on article 5. Most specimens have a pair of setae on dorsal margins of pleonites 1–3 but several specimens have three setae. The length ratio of inner ramus of uropod 3 to outer ramus ranged from 0.2 to 0.3 in both sexes. The number of plumose setae on inner margin of outer ramus of uropod 3 varied from two to eight in males and one to three in females. Ovigerous females have 58 to 175 eggs.

#### Remarks.

*Jesogammarus ikiensis* sp. n. is assigned to the subgenus *Jesogammarus* in having well developed posterior accessory lobe of coxal gills on gnathopod 2 and pereopods 3–5, and pectinate setae on palmar margin of female gnathopod 2. The new species is distinguished from *Jesogammarus fontanus* Hou & Li, 2004, *Jesogammarus hebeiensis* Hou & Li, 2004, *Jesogammarus hinumensis* Morino, 1993, and *Jesogammarus spinopalpus* Morino, 1985 by absence (*vs.* presence) of setae on article 1 of mandibular palp. *Jesogammarus ikiensis* is distinguished from *Jesogammarus mikadoi* Tomikawa, Morino & Mawatari, 2003 by absence (*vs.* presence) of setae on dorsal margin of pereonites 5–7 and two or three (*vs.* more than seven) setae on dorsal margins of pleonites 1–3. *Jesogammarus ikiensis* is distinguished from *Jesogammarus paucisetulosus* Morino, 1984 by medium eye, major axis of eyes 0.4 × height of head (*vs.* small, less than 0.3), posterodistal corner of peduncular article 1 of antenna 1 with a robust (*vs.* slender) seta, posterior margin of peduncular article 2 of antenna 1 with three or four (*vs.* more than five) setae and/or setal bundles, and posterio-marginal setae on peduncular article 4 of antenna 2 shorter (*vs.* longer) than width of article 4 in male;. *Jesogammarus ikiensis* differs from the *Jesogammarus jesoensis* complex including *Jesogammarus fujinoi* Tomikawa & Morino, 2003, *Jesogammarus hokurikuensis* Morino, 1985, *Jesogammarus jesoensis* (Schellenberg, 1937), *Jesogammarus shonaiensis* Tomikawa & Morino, 2003, by two or three (*vs.* more than seven) setae on dorsal margins of pleonites 1–3 and three or four (*vs.* two) setae and/or setal bundles on posterior margin of peduncular article 2 of antenna 1. *Jesogammarus ikiensis* differs from *Jesogammarus ilhoii* Lee & Seo, 1992 by absence (*vs.* presence) of pectinate setae on palmar margin of propodus of male gnathopod 2 and two or three (*vs.* more than ten) setae on dorsal margins of pleonites 1–3.

#### Etymology.

The specific name is from the Latinized Japanese *ikiensis* (of Iki), referring to the type locality of the new species.

#### Distribution.

Known only from Iki Island.

#### Habitat.

River and irrigation ditch.

### Key to species of *Jesogammarus*

Since species of the *Jesogammarus jesoensis* complex including *Jesogammarus fujinoi*, *Jesogammarus hokurikuensis*, *Jesogammarus jesoensis*, *Jesogammarus shonaiensis* are difficult to distinguish from each other due to high variability of morphological characters (Kusano and Ito 2003, Tomikawa unpublished data), only the *Jesogammarus jesoensis* complex is included in the key. In addition, *Jesogammarus naritai* Morino, 1985 is not morphologically distinguishable from *Jesogammarus suwaensis* Morino, 1986 (Tomikawa et al. 2007), and the latter is treated as the same as the former in the key.

**Table:** 

1	Accessory lobes of coxal gills on gnathopod 2 and pereopods 3–5 well developed, both anterior and posterior lobes subequal in length or posterior lobe longer than anterior one; palmar margin of propodus of female gnathopod 2 with pectinate setae	**2** (subgenus *Jesogammarus*)
–	Accessory lobes of coxal gills on gnathopod 2 and pereopods 3–5 weakly developed, anterior and posterior lobes unequal in length, often posterior lobe rudimentary; palmar margin of propodus of female gnathopod 2 without pectinate setae	**10** (subgenus *Annanogammarus*)
2	Article 1 of mandibular palp with setae	**3**
–	Article 1 of mandibular palp without setae	**6**
3	Dorsal margin of pleonites 1–3 each with 1–2 setae; eye large; article 1 of mandibular palp with 1 robust seta; female pereopods densely setose	**Jesogammarus (Jesogammarus) hinumensis Morino, 1993**
–	Dorsal margin of pleonites 1–3 each with more than 4 setae; eye small to medium; article 1 of mandibular palp with 2 or 3 robust setae; female pereopods not densely setose	**4**
4	Peduncular article 1 of antenna 1 with robust seta on posterodistal corner	**Jesogammarus (Jesogammarus) spinopalpus Morino, 1985**
–	Peduncular article 1 of antenna 1 with slender seta on posterodistal corner…5
5	Inner ramus of uropod 3 length 1/4 × outer ramus; inner margin of outer ramus of uropod 3 with 4–6 plumose setae	**Jesogammarus (Jesogammarus) fontanus Hou & Li, 2004**
–	Inner ramus of uropod 3 length 1/3 × outer ramus; inner margin of outer ramus of uropod 3 with about 10 plumose setae	**Jesogammarus (Jesogammarus) hebeiensis Hou & Li, 2004**
6	Dorsal margin of pereonites 1–3 each with 2 long setae	**Jesogammarus (Jesogammarus) mikadoi Tomikawa et al., 2003**
–	Dorsal margin of pereonites 1–3 without setae	**7**
7	Posterior margin of peduncular article 2 of antenna 1 with fewer than five setae and/or setal bundles; posteromarginal setae on peduncular article 4 of antenna 2 shorter than width of article 4 in male; posterodistal corner of peduncular article 2 of antenna 1 with robust seta (occasionally lacking)	**8**
–	Posterior margin of peduncular article 2 of antenna 1 with more than 5 setae and/or setal bundles; posteromarginal setae on peduncular article 4 of antenna 2 longer than width of article 4 in both sexes; posterodistal corner of peduncular article 2 of antenna 1 without robust seta	**Jesogammarus (Jesogammarus) paucisetulosus Morino, 1984**
8	Dorsal margins of pleonites 1–3 each with 2 or 3 setae; posterior margin of peduncular article 2 of antenna 1 with 3 or 4 setae and/or setal bundles	**Jesogammarus (Jesogammarus) ikiensis sp. n.**
–	Dorsal margins of pleonites 1–3 each with more than 7 setae; posterior margin of peduncular article 2 of antenna 1 with 2 setae and/or setal bundles	9
9	Palmar margin of propodus of male gnathopod 2 without pectinate setae	**Jesogammarus (Jesogammarus) jesoensis complex**
–	Palmar margin of propodus of male gnathopod 2 with pectinate setae	**Jesogammarus (Jesogammarus) ilhoii Lee & Seo, 1992**
10	Dorsal margin of pleonite 3 with robust setae; posterior margin of peduncular article 4 and 5 with more than 5 long-setal bundles	**Jesogammarus (Annanogammarus) naritai Morino, 1985**
–	Dorsal margin of pleonite 3 without robust setae; posterior margin of peduncular article 4 and 5 with less than 3 short-setal bundles	**11**
11	Posterodistal corner of bases of pereopods 5–7 with long setae	**Jesogammarus (Annanogammarus) annandalei (Tattersal, 1922)**
–	Posterodistal corner of bases of pereopods 5–7 without short setae	**12**
12	Dorsal margins of pleonites 1–3 each with 2–4 setae	**Jesogammarus (Annanogammarus) fluvialis Morino, 1985**
–	Dorsal margins of pleonites 1–3 each with more than 10 setae	**13**
13	Posterodistal corner of peduncular article 1 of antenna 1 with robust seta; palmar margin of propodus of female gnathopod 2 with simple setae only	**Jesogammarus (Annanogammarus) koreanus Lee & Seo, 1990**
–	Posterodistal corner of peduncular article 1 of antenna 1 without robust seta; palmar margin of propodus of female gnathopod 2 with weakly pectinate setae	**Jesogammarus (Annanogammarus) debilis Hou & Li, 2005**

## Supplementary Material

XML Treatment for Jesogammarus (Jesogammarus) ikiensis
